# A 90 min Daytime Nap Opportunity Is Better Than 40 min for Cognitive and Physical Performance

**DOI:** 10.3390/ijerph17134650

**Published:** 2020-06-28

**Authors:** Omar Boukhris, Khaled Trabelsi, Achraf Ammar, Raouf Abdessalem, Hsen Hsouna, Jordan M. Glenn, Nick Bott, Tarak Driss, Nizar Souissi, Omar Hammouda, Sergio Garbarino, Nicola Luigi Bragazzi, Hamdi Chtourou

**Affiliations:** 1 “Physical Activity, Sport and Health” Research Unit, UR18JS01, National Sport Observatory, Tunis 1003, Tunisia; omarboukhris24@yahoo.com (O.B.); raoufabdesalem18@gmail.com (R.A.); hsen.hsouna92@gmail.com (H.H.); n_souissi@yahoo.fr (N.S.); h_chtourou@yahoo.fr (H.C.); 2 High Institute of Sport and Physical Education of Sfax, University of Sfax, Sfax 3000, Tunisia; trabelsikhaled@gmail.com (K.T.); omar.hammouda@parisnanterre.fr (O.H.); 3 Research Laboratory: Education, Motricity, Sport and Health, EM2S, LR19JS01, High Institute of Sport and Physical Education of Sfax, University of Sfax, Sfax 3000, Tunisia; 4 Institute of Sport Science, Otto-von-Guericke University Magdeburg, 39106 Magdeburg, Germany; Ammar.achraf@ymail.com; 5 Exercise Science Research Center, Department of Health, Human Performance and Recreation, University of Arkansas, Fayetteville, AR 72701, USA; jordan@neurotrack.com; 6 Neurotrack Technologies, 399 Bradford St, Redwood City, CA 94063, USA; nick@neurotrack.com; 7 Clinical Excellence Research Center, Department of Medicine, Stanford University School of Medicine, Stanford, CA 94305, USA; 8 Interdisciplinary Laboratory in Neurosciences, Physiology and Psychology: Physical Activity, Health and Learning (LINP2-2APS), UFR STAPS, UPL, Paris Nanterre University, 92000 Nanterre, France; tarak.driss@parisnanterre.fr; 9 Research Laboratory, Molecular Bases of Human Pathology, LR12ES17, Faculty of Medicine, Sfax 3000, Tunisia; 10 Department of Neuroscience, Rehabilitation, Ophthalmology, Genetics, Maternal and Child Health (DINOGMI), University of Genoa, 16132 Genoa, Italy; sgarbarino.neuro@gmail.com; 11 Department of Health Sciences (DISSAL), Postgraduate School of Public Health, University of Genoa, 16132 Genoa, Italy; 12 Department of Mathematics and Statistics, Laboratory for Industrial and Applied Mathematics (LIAM), York University, Toronto, ON M3J 1P3, Canada

**Keywords:** siesta, sport, muscle soreness, mood, attention, fatigue

## Abstract

This study examined the effects of different nap durations on attention and physical performance as well as mood states, sleepiness, perceived exertion (RPE), recovery (PRS), and muscle soreness (DOMS) in trained men. Fourteen amateur team sport players (age: 20.3 ± 3.0 years, height: 173.1 ± 6.7 cm, body-mass: 68.1 ± 6.6 kg) performed a maximal voluntary isometric contraction (MVIC) test, 5-m shuttle run, and the digit-cancellation (i.e., attention) test after a no-nap (N0) and 40-min (N40) and 90-min (N90) of nap opportunities. Subjective measurement of mood states, RPE, PRS and DOMS were determined. Compared to N0, both nap durations enhanced attention, MVIC, total distance (TD), and higher distance (HD) (*p* < 0.001), with a higher gain after N90 compared to N40 for attention (Δ = +3), MVIC (Δ = +30 N) and TD (Δ = +35 m) (*p* < 0.001). Total mood scores were better after N40 and N90 compared to N0 (*p* < 0.05), with lower scores after N90 compared to N40 (*p* < 0.05). DOMS and RPE scores were significantly lower and PRS was significantly higher after N40 and N90 compared to N0 and after N90 compared to N40 (*p* < 0.05). Although both nap opportunity durations were beneficial, N90 was better than N40 for improving physical performances and attention as well as the perception of recovery, reducing fatigue perception, muscle soreness, and negative mood states.

## 1. Introduction

A major factor reported to significantly disrupt or impair performance outcomes is sleep [1]. For trained athletes, sleep perturbations could be related to late-night competition [2], nightmares before competition [3], and/or early morning training sessions [4]. Sleep is essential for the recovery process due to its physiological and psychological reparative effects [1]. Athletes are frequently exposed to high-intensity training and competition programs and they require more sleep than the general population due to increased mental and physical demands [1]. As a result, daytime napping has been used as a strategy to improve athletes’ sleep quality and quantity [5].

Napping is a recovery period defined as a propensity to sleep in response to the post lunch dip process, which was associated with reductions in core temperature and vigilance and an increase in the tendency to sleep [6]. More importantly, sleepiness [7,8], mood states [9], psychomotor [10], cognitive [7,9,11], and physical performances [11,12,13,14,15,16] have been positively associated with napping. Following a normal night sleep, it has been shown that (i) a 25-min nap opportunity enhanced performance during the 5-m shuttle run test (5mSRT) [12], (ii) a 25-min, 35-min and 45-min nap opportunity decreased subjective fatigue, sleep, and stress [11] and increased physical performance during the 5mSRT [13], (iii) a 35-min and 45-min nap opportunity enhanced 5 jump performance [11], and (iv) a 45-min [11] and 90-min [10] nap opportunity improved attention estimated by the digit cancelation test (DCT).

After partial sleep deprivation, 30-min of napping improved sprint performance and alertness and decreased sleepiness [16] and 20-min and 90-min of napping enhanced repeated sprint performance [15,17]. While recommending athletes to add a nap during the day appears reasonable, there is little empirical evidence as to what nap duration is best for improving physical and cognitive performance. Hammouda et al. [15] and Romdhani et al. [17] reported that a 90-min nap increased repeated-sprint performance more than a 20-min nap in partially sleep-deprived athletes. After a normal night’s sleep, Boukhris et al. [13] demonstrated that a 45-min nap opportunity was the best duration, i.e., compared to a 25-min and a 35-min nap, for enhancing performance and reducing fatigue during the 5-m shuttle run test (5mSRT). Furthermore, Hsouna et al. [11] showed napping between 35-min and 45-min was more effective than 25-min for improving physical performance and alertness. Although these studies portray the importance of nap opportunities on performance, it is still unknown if an increase in nap duration over 45-min would result in further performance improvements.

Therefore, the purpose of the present study was to examine the effects of different nap opportunity durations (i.e., 40-min vs. 90-min) on sleepiness, mood states, attention, maximal voluntary isometric contraction (MVIC), performance during the 5-m shuttle run test (5mSRT), delayed onset muscle soreness (DOMS), recovery (PRS), and exertion (RPE) in trained men who normally slept. Additionally, a comparison between these two nap opportunities durations (i.e., 40-min vs. 90-min) was examined in the current study. These two durations (i.e., 40-min vs. 90-min) of nap opportunities were chosen in the present study because, based on previous studies, the longest nap duration was 90-min in the studies of Hammouda et al. [15] and Romdhani et al. [17]; whereas the longest ones in the study of Boukhris et al. [13] and Hsouna et al. [11] were only 45-min. We hypothesized that both nap durations would prove beneficial and a greater improvement in attention, physical performance, PRS, and reduction in sleepiness, DOMS, and RPE would be observed after a 90-min nap (N90), compared to a 40-min nap (N40) and no-nap (N0) condition.

## 2. Materials and Methods

### 2.1. Participants

Fourteen amateur team sport players (soccer (*n* = 7), rugby (*n* = 3), handball (*n* = 4) (age: 20.3 ± 3.0 years, height: 173.1 ± 6.7 cm, body-mass: 68.1 ± 6.6 kg) volunteered to participate in this study. Participants were players who played football, rugby or handball on behalf of their college/schools or a local second or third division club. All athletes trained at least 4 days per week for an average of 2-h per day. After receiving a thorough description of the protocol, each volunteer provided written informed consent. The present study was conducted according to the Declaration of Helsinki and the protocol was fully approved by the local Ethics Committee (CPP: 0098/2018). The participants were non-smokers, did not have pathological sleep disorders, and did not consume alcohol.

### 2.2. Experimental Design

The study used a crossover repeated-measures design consisting of three test sessions. After a familiarization session, participants randomly attended three test sessions (i.e., N0, N40, and N90) with at least 72 h in-between. They got into bed at 13:45 h in rooms that were favorable to sleep (i.e., dimly lit and quiet). After 15 min of becoming accustomed to their sleep environment, participants in the nap conditions were asked to take a nap from 14:00 h to (i) 14:40 h for N40 or (ii) 15:30 h for N90. Naps were realized at 14:00 h as this phase is taken naturally after lunch, between 13:00 h and 16:00 h, at a time when vigilance decreases significantly and there are strong feelings of sleepiness [12]. Abdessalem et al. [12] compared three nap times (i.e., 13:00 h, 14:00 h and 15:00 h) and reported that 14:00 h and 15:00 h were the best nap moments for the 5msSRT performance enhancement. As one of the nap duration in the present study was 90 min, and to avoid sleep inertia, the 14:00 h time was selected. For N0 and the two nap opportunity conditions, participants spent the remaining time until 17:00 h reading books, watching videos on television, or playing video games in a comfortable armchair. The night before each experimental test, participants wore GT3X activity monitors on the non-dominant arm (Actigraph, Pensacola, FL, USA) to record sleep patterns and ensure adherence to a consistent sleep-wake schedule. Participants were asked to keep a normal sleep duration (i.e., 7–9 h) throughout the experimental period to avoid any undesirable effect of sleep deprivation. That meant participants were asked to sleep between 22:30 h and 23:00 h and wake up between 07:30 h and 08:15 h. Participants were also asked about their subjective sleep quality for each nap (i.e., N40 and N90) using a scale ranging from “0” (no sleep) “5” (some sleep with some interruptions) to “10” (uninterrupted, deep sleep throughout) [16]. In addition to that, the Stanford Sleepiness Scale (SSS) was used to give a subjective rating of sleepiness before (i.e., 13:45 h) and after each experimental condition (i.e., 15:30 h for N0, 14:40 h for N40 and 15:30 h for N90). The SSS is a 7-point scale ranging from “1” (high activeness) to “7” (high tiredness). More than 1 h was allowed for participants to overcome any sleep inertia that might have occurred after napping. In fact, 30 min has been shown as sufficient to overcome sleep inertia [15,16,17].

During all sessions (i.e., N0, N40 and N90), at 16:45 h, participants began by answering the profile of mood states (POMS) questionnaire and doing the digit cancellation test. The POMS is a self-report questionnaire consisting of 65 adjectives designed to assess six states (i.e., tension, depression, anger, vigor, fatigue, confusion). Responses to each item range from “0” (Not at all) to “4” (Extremely), with higher scores indicating a more negative mood [18]. The digit cancellation test (DCT) consisted of crossing-off the given target numbers (i.e., the number composed by three digits) on a sheet of randomly arranged possibilities in 1 min to estimate the attention of the participant [19]. The sum of the correct crossed-off numbers was then calculated.

Next, participants completed a 10 min standardized warm up at 17:00 h. This consisted of a 5 min jog at a self-selected comfortable pace, followed by a 5-min series of dynamic stretching (e.g., hip flexion/extension, hip abduction/adduction, butt kicks), and five progressive accelerations. Then participants performed three maximal voluntary isometric contractions (MVIC); participants were instructed to exert maximal voluntary knee extension against the lever arm. The highest MVIC value among the three trials was recorded for analysis. In order to eliminate the confounding effects of fatigue caused by repeated muscle contractions, the realized MVICs were separated by a recovery period of 2 min. After the MVICs, a rest of 5 min was allowed and then the participants performed the 5mSRT. RPE scores were registered immediately after the 5mSRT on a scale ranging from “0” (nothing at all) to “10” (maximal) [20]. Additionally, participants answered the delayed-onset muscle soreness (DOMS) and the perceived recovery status scale (PRS) 3 min after the 5mSRT. The DOMS scale ranging from “1” (no soreness) “5” (sore) to “10” (very sore) is a subjective rating of lower limb muscle soreness [21]. The PRS is an 11-point scale ranging from “0” to “10”, with 0–2 representing “very poorly recovered and with anticipated declined in performance”, 4–6 representing “low to moderately recovered and expected similar performance”, and 8–10 representing “high perceived recovery with expected improvement in performance” [22].

### 2.3. The Actigraphs Registration and Analysis

The night before each experimental test, participants wore GT3X activity monitors on their non-dominant arm (Actigraph, Pensacola, FL, USA) to record their sleep patterns and to ensure adherence to a consistent sleep-wake schedule. The actilife 6 was used to analyze sleep and wake behaviors of the night prior to testing. The Sadeh algorithm was used to calculate sleep parameters due to its overall high accuracy compared to that of polysomnography [23]. Time in bed was calculated via the sleep diary. The difference between bedtime and time awake was manually recorded and cross-validated against actigraphy [24]. A high-level of sensitivity was used for the assessment of sleep-wake patterns in 60-s epochs. The sleep parameters obtained were: bed time, out of bed time, sleep latency, sleep efficiency, total time in bed, and total sleep time.

### 2.4. Maximal Voluntary Isometric Contraction

MVIC of the right leg was measured using an isometric dynamometer (Good Strength, Metitur, Finland) equipped with a cuff attached to a strain gauge at the hip. The angle of the knee was kept at 90° (knee full extension = 0°) while the participant was seated. In order to avoid lateral, vertical, or frontal displacements, safety belts were strapped across the chest, thighs, and hips of the participant.

### 2.5. The 5-m Shuttle Run Test

With a 35-s recovery between repetitions, participants were instructed to perform six 30-s maximal shuttle sprints of increasing distances: 5 m, 10 m, 15 m, 20 m, etc. [25]. The distance covered in each repetition was registered and the greatest distance (highest distance (HD)) during a 30-s shuttle, the total distance (TD) covered during the six 30-s shuttles, and the fatigue index (FI) were calculated. FI was calculated as follows:

(1) $$FI\;\left(\%\right)=\frac{\left(shuttle\;1+shuttle\;2\right)-\left(shuttle\;5+shuttle\;6\right)}{shuttle\;1+shuttle\;2}\times 100$$

### 2.6. Statistical Analysis

Data are presented as means ± standard deviation (SD) and were analyzed using the Statistica software (StatSoft, version 12, Paris, France).

G * power software (version 3.1.9.2; Kiel University, Kiel, Germany) [26] was used to calculate the required sample size. Values for α were set at 0.05 and power at 0.80. Based on the study of Boukhris et al. [13] and discussions between the authors, effect size was estimated to be 0.65. The required sample size was six for physical performance. Concerning cognitive performance, based on the study of Hsouna et al. [11] and discussions between the authors, Cohen d effect size was estimated to be 0.39. The required sample size was thirteen for cognitive performance.

Normality of the distributions was confirmed using the Shapiro–Wilk test. Data of bed time, out of bed time, sleep latency, total time in bed, total sleep time, MVIC, HD, TD, FI, attention, confusion, and vigor were normally distributed. Therefore, a one-way ANOVA (Nap) was performed for these parameters and post hoc comparisons were made using the Bonferroni test. However, when normality was not confirmed, a Friedman nonparametric analysis of variance (ANOVA) was used for sleep efficiency, sleepiness perception, DOMS, PRS, RPE, tension, depression, anger, and fatigue. Pairwise comparisons were conducted using a Wilcoxon test.

Effect sizes for the normally distributed variables were calculated as partial eta-squared (*η_p_*^2^). Partial eta-squared values of 0.01, 0.06 and 0.13 represented small, moderate, and large effect sizes, respectively. For the non-normally distributed variables, effect sizes were estimated by the Kendall’s coefficient of concordance.

To explain possible changes in physical outcomes in responses to the different nap durations, the relationships between variables was examined using Pearson for data normally distributed or Spearman for data not normally distributed.

Significance for all analyses was set at *p* < 0.05. Exact p values are provided; results given as “0.000” in the statistics output have been reported as “<0.0005”. Additionally, the gain or decrease for all parameters (Δ) was calculated.

## 3. Results

### 3.1. Sleep Parameters

The sleep parameters (i.e., bed time, out of bed time, sleep latency, sleep efficiency, total time in bed and total sleep time) were similar during the night preceding each condition of the study (Table 1).

### 3.2. Maximal Voluntary Isometric Contraction

MVIC values are presented in Table 2. The post hoc analysis revealed that MVIC was higher (*p* < 0.0005, Δ = +43) after N40 and higher (*p* < 0.0005, Δ = +74) after N90 compared to N0. In addition, MVIC after N90 was higher than N40 (*p* < 0.0005, Δ = +30).

### 3.3. Higher Distance (HD)

HD values are presented in Table 2. The post hoc analysis showed that HD was 10 m higher (*p* < 0.0005) after N40 and 13 m higher (*p* < 0.0005) after N90 compared to N0.

### 3.4. Total Distance (TD)

TD values are presented in Table 2. The post hoc analysis showed that TD was 55 m higher (*p* < 0.0005) after N40 and 89 m higher (*p* < 0.0005) after N90 compared to N0. In addition, TD after N90 was 35 m higher than N40 (*p* = 0.04).

### 3.5. Fatigue Index (FI)

FI values are presented in Table 2. The post hoc analysis showed that FI was lower after N90 compared to N0 (*p* = 0.001, Δ = −5).

### 3.6. The Attention Scores

Attention scores are presented in Table 2. The attention scores were higher (*p* < 0.0005, Δ = +6) after N40 and higher (*p* < 0.0005, Δ = +9) after N90 compared to N0. In addition, the attention scores after N90 were higher than N40 (*p* = 0.001, Δ = +3).

### 3.7. Perception of Sleepiness

**Figure 1:**
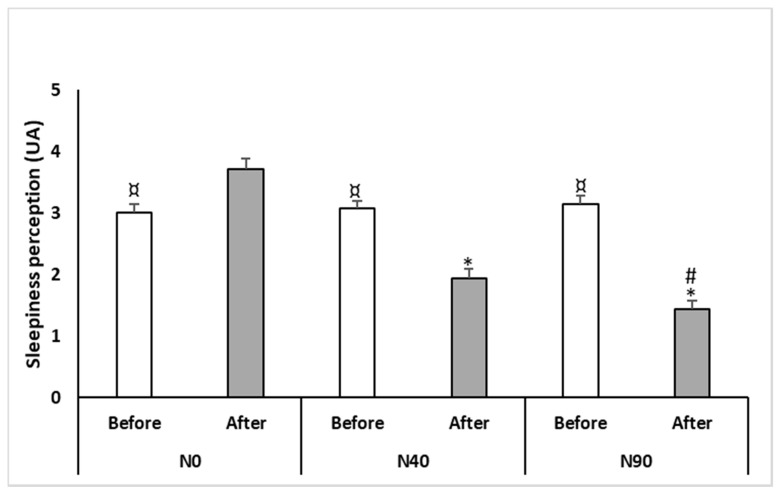
Perception of sleepiness was recorded before and after the 40-min (N40) and the 90-min (N90) of nap opportunities and the no-nap condition (N0). ¤ Significant difference compared to after the time of each condition; * Significant difference compared to the N0; # Significant difference compared to N40.

A Friedman test conducted on sleepiness perception reported a significant effect (test = 57.95, *p* < 0.0005, Kendall’s W = 0.82); an increase of sleepiness perception was observed when compared to before the time of N0 (*p* = 0.01, Δ = 0.7) (Figure 1). However, sleepiness perception was lower after N40 (*p* = 0.001, Δ = −1.1) and N90 (*p* = 0.0009, Δ = −1.7) compared to N0 (Figure 1).

In addition, sleepiness perception recorded after both nap opportunities was lower than after N0 (*p* = 0.001; Δ = −1.8 for N40 and *p* = 0.0009; Δ = −2.3 for N90). However, N90 resulted in lower sleepiness perception in comparison with N40 (*p* = 0.03 and Δ = −0.5).

### 3.8. Profile of Mood States

The statistical analysis revealed a significant main effect of nap for tension (test = 19.85; *p* < 0.0005; Kendall’s W = 0,70), anger (test = 9.48; *p* = 0.008; Kendall’s W = 0.33), depression (test = 18.56; *p* < 0.0005; Kendall’s W = 0.66), fatigue (test = 24.29; *p* < 0.0005; Kendall’s W = 0.86), vigor (F = 39.06; *p* < 0.0005; n_p_^2^ = 0.75), and the total POMS score (test = 23.67; *p* < 0.0005; Kendall’s W = 0.84).

N40 and N90 improved tension, depression, vigor, fatigue, and total score in comparison with N0 (*p* < 0.05). However, N90 resulted in lower tension, depression, fatigue, and total score in comparison with N40. Contrariwise, anger decreased after N90 in comparison with N0 (*p* = 0.003) and N40 (*p* = 0.009) (Table 3).

However, there was no-significant main effect of a nap on confusion (F = 0.23; *p* = 0.79; n_p_^2^ = 0.01).

### 3.9. Delayed Onset Muscle Soreness

DOMS scores are presented in Table 4. DOMS scores recorded after the 5mSRT were lower after N40 (*p* = 0.02, Δ = −0.7) and lower after N90 in comparison with N0 (*p* = 0.002, Δ = −1.3). However, N90 resulted in lower DOMS in comparison with N40 (*p* = 0.04 and Δ = −0.6).

### 3.10. Perceived Recovery Status Scale 

PRS score are presented in Table 4. PRS scores recorded after the 5mSRT were higher after N40 (*p* = 0.005, Δ = +1) and higher after N90 (*p* = 0.002, Δ = +1.6) in comparison with N0. N90 resulted in higher PRS compared to N40 (*p* = 0.02 and Δ = +0.6).

### 3.11. Rating of Perceived Exertion Scale

RPE scores are presented in Table 4. RPE scores recorded after the 5mSRT were lower after N40 (*p* = 0.005, Δ = −1.1) and lower after N90 in comparison with N0 (*p* = 0.0009, Δ = −1.9). N90 resulted in lower RPE in comparison with N40 (*p* = 0.005 and Δ = −0.9).

### 3.12. Correlation

#### 3.12.1. N0 Compared to N40

The correlation analysis is presented in Table 5. The MVIC was correlated with sleepiness perception and vigor. HD was correlated with sleepiness perception, fatigue, vigor, and RPE. TD was correlated with sleepiness perception, fatigue, vigor, RPE, PRS and DOMS. Attention measured by DCT was correlated with sleepiness perception, fatigue and vigor.

#### 3.12.2. N0 Compared to N90

The correlation analysis is presented in Table 6. The MVIC was correlated with sleepiness perception, fatigue and vigor. HD was correlated with sleepiness perception, fatigue, vigor, RPE, PRS and DOMS. TD was correlated with sleepiness perception, fatigue, vigor, RPE, PRS and DOMS. FI was correlated with sleepiness perception, fatigue, vigor, RPE, PRS and DOMS. Attention measured by DCT was correlated with sleepiness perception, fatigue and vigor.

#### 3.12.3. N40 Compared to N90

The correlation analysis is presented in Table 7. The MVIC was correlated with sleepiness perception, fatigue and vigor.

TD was correlated with sleepiness perception, fatigue, vigor, RPE, PRS and DOMS.

Attention measured by DCT was correlated with sleepiness perception, fatigue and vigor.

## 4. Discussion

The present study assessed the effect of different nap opportunity durations (i.e., N40 and N90) on MVIC, physical performance during the 5mSRT, attention, sleepiness, and mood states following a normal sleep night. The main findings of the current study were that both nap opportunity durations positively affected MVIC, physical performance during the 5mSRT, attention, sleepiness, and mood states. In general, greater beneficial effects were observed after N90 in comparison with N40.

There were no significant differences in sleep the night before each of the three experimental conditions (i.e., 566 min in N0, 515.4 min in N40 and 525.8 min in N90). Furthermore, each night’s sleep duration represented sleep quantity (i.e., 8–9 h) consistent with the 7–9 h sleep recommendations [27]. Previous studies are limited by the lack of an objective measurement of the previous night’s sleep [11,12,13,15,16,17,28], which may confound their results. In this study, we confirmed total time sleep before each condition via the use of actigraphy, similar to previous work [14,29,30].

Our results were consistent with those reported by other studies confirming the beneficial effect of napping on cognitive [11,14,16] and physical performance [11,12,13,15,16,17,28,29,31,32], independent of the previous night’s sleep. For example, following a normal night sleep, it has been shown that (i) a 25-min nap opportunity enhanced performance during the 5mSRT [12], (ii) a 25-min, 35-min and 45-min nap opportunity increased physical performance during the 5mSRT [13], (iii) a 35-min and 45-min nap opportunity enhanced 5 jump performance [11], and (iv) a 45-min [11] and 90-min [10] nap improved attention estimated by the digit cancelation test (DCT). After partial sleep deprivation, 30-min of napping improved sprint performance and alertness [14,16], and 20-min and 90-min of napping enhanced repeated sprint performance [15,17].

However, Petit et al. [33] revealed a 20-min nap after normal or 5-h sleep did not improve physical performance during the Wingate test. The contradiction between these results could be related to the nap duration. In fact, previous studies have reported that the length of a nap affects its efficacy for improving physical performance [11,13,15,17].

Many factors are positively related to daytime napping, such as a reduction in sleepiness [16,17]. In the current study, sleepiness perception was affected positively by N40 and N90, which could explain the improvement in cognitive, as well as physical performance. To further support these findings, changes in attention, HD, TD, and MVIC were negatively correlated with changes in sleepiness perception for N40 and N90. Thus, decreases in sleepiness perception was associated with an increase in cognitive and physical performances.

In addition, mood states were positively related to napping [9,17]. In the present study, mood states estimated by the POMS (i.e., tension, depression, vigor, fatigue and total mood score) were positively affected by N40 and N90, which may explain improvements in physical and cognitive performance. The correlation analysis supports this assumption; both N40 and N90 demonstrated positive correlations with changes in vigor, physical performance, and attention. This suggests that increases in vigor may be important for subsequent improvements in physical and cognitive performance. Additionally, a negative correlation between changes in fatigue, physical, and cognitive performance indicates that decreases in fatigue improved HD, TD, MVIC, and attention. Another possible explanation of the enhancement of physical performance during the 5mSRT is the reduction of RPE [13,17] and DOMS, as well as increases in PRS. The present study revealed significant correlations between the (i) reduction of RPE (in N40 and N90) and DOMS (in N90) and increase of PRS (in N90) and (ii) improvement of HD and TD during the 5mSRT. Additionally, TD increase was related to the enhancement of PRS and the reduction of DOMS after N40. Furthermore, the enhancement of physical and cognitive performance could be related to the amount of slow wave sleep contained in each nap duration [11,13,15,17]

Concerning the most beneficial nap duration, N90 appears to be the best nap duration for improving attention, MVIC, and TD during the 5mSRT. In this context, MVIC increased by 4% after N90 compared to N40. TD increased by 4% or 35 m after N90 compared to N40. Similarly, attention determined by the DCT increased by 3% after N90 compared to N40. Thus, it could be posited that participants performed better after N90 due to increased alertness. A significant decrease by 54% in sleepiness perception was observed after N90 in comparison with N40. The current study also reported a significant correlation for change (i.e., between N90 and N40) between physical cognitive performance (i.e., MVIC, TD) and sleepiness perception. In the same way, it has been suggested that a long afternoon nap may be comparable to a night’s sleep in terms of sleep quality, whereas it may enhance greater autonomic arousal after awakening than that in the morning [34]. The results of the present study were consistent with those of Hammouda et al. [15] who reported that a nap duration of 90-min compared to 20-min was better for improving physical performance. Conversely, Hsouna et al. [11] reported that naps of 35-min and 45-min were better than 25-min for improving the 5-jump performance. Likewise, the most beneficial nap duration in the study of Boukhris et al. [13] was 45-min compared to 25-min and 35-min for enhancing performance during the 5-m shuttle run test. Recently, Romdhani et al. [17] confirmed the hypothesis that a 90-min nap is better for enhancing physical performance in comparison with 20-min. Thus, the longer nap had stronger enhancing effects on cognitive and physical performance.

Furthermore, it has been reported that mood states and physical performance have a direct relationship with sleep quality and quantity [35,36]. In this context, mood states estimated by the POMS (i.e., tension, depression, anger and fatigue) were significantly lower after N90 compared to N40. In agreement with present findings, Romdhani et al. [17] revealed N90 improved mood states more than a 20-min nap. Thus, the higher enhancement of MVIC, TD, and FI during the 5mSRT after N90 could be explained by a reduction in fatigue and an increase in vigor. Additionally, the present study revealed a significant correlation between (i) fatigue and vigor and (ii) physical performance (i.e., MVIC, TD) were observed for changes between N90 and N40, and between (i) fatigue and vigor and (ii) FI for changes between N90 and N0.

N90 resulted in higher PRS compared to N40, which could explain the greater improvement in TD and FI during the 5mSRT. TD during the 5mSRT was positively correlated with PRS for changes between N90 and N40, leading to an increase in the perception of recovery enhance TD.

Additionally, the current study demonstrated that DOMS recorded after the 5mSRT was lower after N90 in comparison with N40. Therefore, we speculate that better improvements in TD and FI during the 5mSRT (after N90) may be related to lower scores of DOMS. In support of this idea, a significant negative correlation was observed between TD and DOMS for change between N90 and N40. A significant correlation between FI and DOMS for change between N90 and N0 was also reported.

Moreover, N90 resulted in lower RPE scores compared to N40, supporting the results of Boukhris et al. [13] and Romdhani et al. [17] who reported that lower RPE scores were greater associated with long nap durations than short nap durations. In this context, for changes between N90 and N40, there are significant negative correlations between TD and RPE scores. Additionally, a significant positive correlation was observed between FI and RPE scores for changes between N90 and N0.

In the present study, for the 5mSRT, N90 resulted in higher TD compared to N40, however, there were no significant differences between N40 and N90 for HD. This contradictory finding could be explained by the physical and physiological determinants related to HD and TD. In this context, HD could be an indicator of alactic or adenosine triphosphate and phosphocreatine (ATP-PCr) capacity [12]. However, TD is likely related to aerobic power and the ability to recover quickly between sprints [12]. Thus, it could be possible that N90 has more influence on aerobic power and metabolic recovery. FI reflected the fatigue induced by the 5mSRT by the calculation of fatigue between one or two first sprints compared to one or two last sprints. The results indicated that FI was affected positively only by the N90, which could explain the higher improvement of TD after N90 in comparison with N40 and confirms the positive relation between N90 and aerobic power and the ability to recover quickly between sprints. 

N90, in comparison with N40, has a greater duration of slow wave sleep. Indeed, slow wave sleep is known as a recovery period for daily metabolism [37,38], which restores physical damage (i.e., stress to bones, muscles, tissues, and organs) and reduces stress and anxiety. It metabolizes fats and carbohydrates, and fortifies the immune system [39]. For this reason, a greater metabolic recovery could be generated by the N90 [17]. Additionally, N90 could represent a full sleep cycle and consequently the presence of rapid eye movement (REM) sleep stages. Moreover, Cai et al. [40] suggested that REM sleep improves muscular efficiency. Thus, higher improvements of physical performance (i.e., MVIC and 5mSRT) after N90 could be related to REM sleep.

Another explanation of higher enhancements of MVIC, TD, and FI after N90 in comparison with N40, is that N90 resulted in higher waking cortisol levels [17]. Indeed, cortisol boosts energy supply [17], which could clarify greater enhancements of MVIC, TD and FI during the 5mSRT after N90.

A limitation of the current study was the lack of physical activity and training data before the experimental sessions as these variables may influence physical and cognitive assessment. In addition, sleep onset latency was self-estimated using a sleep diary; however, it should be noted that previous research indicates self-estimated sleep onset latency is often overestimated when measured in this manner [24]. Another limitation of the present study was the lack of measurement of napping with polysomnography. Actigraphy is not effective to assess a short nap because it estimates sleep time by recording the body’s movements; during a short nap, it may be possible that the participant does not move but is not sleeping. Thus, a measurement of the nap using polysomnography equipment would be required to record the nap time during each condition. While napping is often seen as a response to restore in case of insufficient nocturnal sleep, the current study shows a nap effect in healthy athletes with normal sleep. However, these results could not be generalized to all populations. Also, intra-individual variations in attention, mood and fatigue may confound the post-nap differences between conditions. Thus, further studies should examine these parameters before and after the nap opportunity.

## 5. Conclusions

Following a normal night sleep, consistent with the 7–9 h sleep recommendations [27], a post-lunch nap opportunity improved physical and cognitive performance. The performance increases induced by N40 and N90 were explained by a decrease in sleepiness and perceived exertion and muscle soreness and were related to improvement of mood states and perceived recovery. However, compared to N40, N90 was the most beneficial nap duration for improving physical and cognitive performance, mood states and perceived recovery, and for reducing perceived exertion and muscle soreness and sleepiness. These findings are important in healthy athletes, and are not generalizable to other populations.

From a practical point of view, coaches and athletes should plan periods of nap (with longer possible durations) before intense afternoon training sessions or late afternoon competition.

## Figures and Tables

**Table 1:** Sleep parameters of the night preceding each condition (i.e., no-nap (N0), 40 min (N40), and 90 min (N90) nap opportunity), and the subjective sleep scale scores of each nap duration.

	No-Nap	N40	N90
Bed time (hh:min)	10:31 ± 00:03	10:45 ± 00:03	10:37 ± 00:03
Out of bed time (hh:min)	08:04 ± 00:09	07:25 ± 00:05	07:40 ± 00:06
Sleep latency (min)	9 ± 5	8 ± 5	8 ± 5
Sleep efficiency (%)	98.9 ± 2.2	99.3 ± 0.6	99.7 ± 0.6
Total time in bed (min)	570.9 ± 144.8	519.1 ± 80.3	527.8 ± 114.5
Total sleep time (min)	566.0 ± 149.0	515.4 ± 79.2	525.8 ± 111.1
Sleep quality estimation of nap (a.u)	-	07.1	07.4

**Table 2:** Attention, maximal voluntary isometric contraction (MVIC), higher distance (HD), total distance (TD) and fatigue index (FI) recorded during the 5-m shuttle run test in the 40-min (N40) and the 90-min (N90) of nap opportunities and the no-nap condition (N0).

	N0	N40	N90	ANOVA	*p* Value	ƞ_p_^2^
Attention scores	79 ± 11	85 ± 12 *	87 ± 13 *,#	F = 86.47	<0.0005	0.86
MVIC (N)	769 ± 94	812 ± 100 *	843 ± 102 *,#	F = 71.05	<0.0005	0.84
HD (m)	129 ± 6	139 ± 11 *	142 ± 13 *	F = 14.39	<0.0005	0.52
TD (m)	704 ± 37	759 ± 71 *	793 ± 64 *,#	F = 23.37	<0.0005	0.64
FI (%)	15 ± 4	12 ± 4	10 ± 3 *	F = 7.70	0.002	0.37

* Significant difference compared to the N0; # Significant difference compared to N40.

**Table 3:** Representation of the results of the profile of mood states recorded after the no-nap condition (N0) and the 40-min (N40) and the 90-min (N90) nap opportunities.

	N0	N40	N90
Tension (a.u)	10.2 ± 3.2	7.4 ± 2.7 *	6.8 ± 3.5 *,#
Depression (a.u)	10.1 ± 8.5	7.9 ± 8.0 *	6.4 ± 7.2 *,#
Anger (a.u)	11.4 ± 8.6	9.6 ± 6.0	6.9 ± 5.5 *,#
Vigor (a.u)	14.1 ± 5.0	16.9 ± 4.2 *	18.1 ± 5.2 *
Fatigue (a.u)	6.0 ± 4.8	4.1 ± 4.0 *	3.4 ± 3.9 *,#
Confusion (a.u)	7.4 ± 3.9	5.9 ± 4.8	5.4 ± 4.4
Total score (a.u)	30.9 ± 19.3	18.0 ± 19.6 *	10.9 ± 18.0 *,#

Note: a.u arbitrary units; * Significant difference compared to N0; # Significant difference compared to N40.

**Table 4:** Delayed onset muscle soreness (DOMS), perceived recovery status scale (PRS), and rating of perceived exertion scale (RPE) recorded in the 40-min (N40) and the 90-min (N90) of nap opportunities and the no-nap condition (N0).

	N0	N40	N90	Freidman Test	*p* Value	Kendall’s W
DOMS (a.u)	7 ± 1	6 ± 1 *	5 ± 1 *,#	test = 16.73	<0.0005	0.59
PRS (a.u)	3 ± 1	4 ± 1 *	5 ± 1 *,#	test = 19.27	<0.0005	0.68
RPE (a.u)	8 ± 1	7 ± 1 *	6 ± 1 *,#	test = 24.00	<0.0005	0.85

Note: a.u arbitrary units; * Significant difference compared to the N0; # Significant difference compared to N40.

**Table 5:** Correlations between selected parameters for changes between N40 and N0.

		Sleepiness Perception	Vigor	Fatigue	RPE	PRS	DOMS
Attention	r	−0.54	0.64	−0.76	NA		
	*p*	=0.04	=0.01	=0.002			
MVIC	r	−0.74	0.76	−0.49	NA		
	*p*	=0.002	=0.001	=0.07			
HD	r	−0.69	0.61	−0.59	−0.60	0.50	−0.50
	*p*	=0.006	=0.01	=0.02	=0.02	=0.06	=0.06
TD	r	−0.62	0.67	−0.60	−0.63	0.69	−0.60
	*p*	=0.01	=0.008	=0.02	=0.01	=0.005	=0.02

**Table 6:** Correlations between selected parameters for changes between N90 and N0.

		Sleepiness Perception	Vigor	Fatigue	RPE	PRS	DOMS
Attention	r	−0.70	0.57	−0.72	NA		
	*p*	=0.004	=0.03	=0.004			
MVIC	r	−0.65	0.69	−0.56	NA		
	*p*	=0.01	=0.006	=0.03			
HD	r	−0.86	0.58	−0.60	−0.70	0.53	−0.68
	*p*	<0.0005	=0.02	=0.02	=0.004	=0.04	=0.007
TD	r	−0.81	0.69	−0.62	−0.74	0.69	−0.55
	*p*	<0.0005	=0.005	=0.01	=0.002	=0.006	=0.04
FI	r	0.58	−0.59	0.65	0.58	−0.64	0.58
	*p*	=0.02	=0.02	=0.01	=0.02	=0.01	=0.02

**Table 7:** Correlations between selected parameters for changes between N90 and N40.

		Sleepiness Perception	Vigor	Fatigue	RPE	PRS	DOMS
Attention	r	−0.55	0.54	−0.71	NA		
	*p*	=0.03	=0.04	=0.003			
MVIC	r	−0.61	0.60	−0.56	NA		
	*p*	=0.01	=0.02	=0.03			
TD	r	−0.59	0.64	−0.60	−0.62	0.69	−0.69
	*p*	=0.02	=0.01	=0.02	=0.01	=0.006	=0.006
